# The Morton Testimonial

**Published:** 1865-12

**Authors:** 


### THE MORTON TESTIMONIAL.

Dr. William T. Morton, whose good fortune it was first publicly to demonstrate the practicability and safety of using ether as an anæsthetic agent, recently visited this city soliciting money to reimburse him for expenses he claims to have incurred in disseminating a knowledge of the discovery, and establishing the practice of using this remedy for preventing pain. Since 1846, Dr. Morton has been engaged in one unremitting struggle to secure to himself, the most ample pecuniary reward, that he hoped might flow from his connection with this felicitous discovery. From year to year, he has importuned Congress for an appropriation from the public treasury—a number of times engineering his bill through one house only to loose it in the other—until at length the fruition which before had eluded his grasp, seemed within his reach, for a bill giving to him $200,000, having passed one branch of Congress, was lost in the other, by a single vote. This defeat in the very moment of victory, was the last straw that broke the camel's back, and giving up all hope of being rewarded from the national treasury, he determined to make a direct appeal to the people, and endeavor to obtain the $200,000 through the somewhat complex machinery of the "Morton Testimonial." The anæsthetic agents are conceded by all to be among the most precious boons that a kind Providence has bestowed on suffering mortals, and that Congress should persistently refuse to reward Dr. Morton, would seem to imply great national injustice. Our good name as a people, requires an explanation of this apparent neglect. Dr. Morton's misfortunes of which he complains, legitimately flow from his own acts. He seems to be so lacking in native dignity of character as never to have viewed the great discovery in any other than a pecuniary light. This inspired him first to attempt to keep the remedy a secret, under the name "letheon," and then to obtain for anæsthesia a patent, designed to prevent the freedom of its use. He has, too, very unjustly and unwisely attempted to suppress the claims of Wells, Jackson and Long, to any participation in the honors due to this discovery. To have spent money to secure congressional action in his behalf was folly, and if he has done so to induce the world to accept the proffered blessing, it was unnecessary. No such effort was ever made in the west, and certainly in no place was the agent more speedily or generally used than here. Indeed in no part of the world did the remedy meet sufficient opposition seriously to retard, much less to endanger its universal acceptance and approval. The final resort to a "testimonial," is a novelty—not so much in outline and substance—as in the manner of its execution. We had been accustomed to look upon such movements as the spontaneous pulsations of the popular heart, quickened by a sense of gratitude and obligation, and had supposed that the recipients of these demonstrations, if not passive observers of such movements, at least lent them only clandestine aid and encouragement. But Dr. Morton has reversed all this and boldly presents himself in the attitutude of a needy supplicant for the nation's gratitude, and specifying that it be rendered in a substantial pecuniary form.

It is painful to witness such indelicacy, and especially when it is exhibited by one who is even nominally a member of the profession of medicine, and who has indissolutely associated his name with the origin of anæsthesia. We think Congress ought to have made a generous appropriation, to be divided among those who are admitted to have been jointly instrumental in perfecting this discovery, and since it has failed to do so, there seems a propriety in individuals supplying the omission, and we are glad to know that in this manner Dr. Morton has, in the eastern cities, received munificent tokens of popular favor. But in the west, this is asked in a manner most likely to defeat its object. The germ of mendicity is found in the very dregs of the mind's endowments, and does not harmonize with the stalwart self-relying spirit of our people, and no unmaimed person can ask alms of them without, in some degree, forfeiting their respect. We know nothing of the pecuniary results of Dr. Morton's visit to this city, but should not be surprised some day to read the history of his experience here, added as a supplemental chapter to the work he published some years since, in which are narrated the trials and tribulations of a public benefactor.

Accidental Amputation of the Tongue.—A boy, sitting on the shaft of a wagon, was jerked off, and his face crushed by one of the wheels. He received a compound fracture of the inferior maxilla, and the edge of the bone turning inward completely amputated the tongue at its base, with the exception of a few shreds of tissue. There was free but not troublesome hemorrhage. He gradually recovered, and when he left the hospital, his method of speech, though very confused and mumbling, was by a little trouble, intelligible.—*London Lancet.*

